# Monitoring for myelopathic progression with multiparametric quantitative MRI

**DOI:** 10.1371/journal.pone.0195733

**Published:** 2018-04-17

**Authors:** Allan R. Martin, Benjamin De Leener, Julien Cohen-Adad, Sukhvinder Kalsi-Ryan, David W. Cadotte, Jefferson R. Wilson, Lindsay Tetreault, Aria Nouri, Adrian Crawley, David J. Mikulis, Howard Ginsberg, Eric M. Massicotte, Michael G. Fehlings

**Affiliations:** 1 University of Toronto, Toronto, Ontario, Canada; 2 Polytechnique Montreal, Montreal, Quebec, Canada; Oslo Universitetssykehus, NORWAY

## Abstract

**Background:**

Patients with mild degenerative cervical myelopathy (DCM) are often managed non-operatively, and surgery is recommended if neurological progression occurs. However, detection of progression is often subjective. Quantitative MRI (qMRI) directly measures spinal cord (SC) tissue changes, detecting axonal injury, demyelination, and atrophy. This longitudinal study compared multiparametric qMRI with clinical measures of progression in non-operative DCM patients.

**Methods:**

26 DCM patients were followed. Clinical data included modified Japanese Orthopedic Association (mJOA) and additional assessments. 3T qMRI data included cross sectional area, diffusion fractional anisotropy, magnetization transfer ratio, and T2*-weighted white/grey matter signal ratio, extracted from the compressed SC and above/below. Progression was defined as 1) patients’ subjective impression, 2) 2-point mJOA decrease, 3) ≥3 clinical measures worsening ≥5%, 4) increased compression on MRI, or 5) ≥1 of 10 qMRI measures or composite score worsening (p < 0.004, corrected).

**Results:**

Follow-up (13.5 ± 4.9 months) included mJOA in all 26 patients, MRI in 25, and clinical/qMRI in 22. 42.3% reported subjective worsening, compared with mJOA (11.5%), MRI (20%), comprehensive assessments (54.6%), and qMRI (68.2%). Relative to subjective worsening, qMRI showed 100% sensitivity and 53.3% specificity compared with comprehensive assessments (75%, 60%), mJOA (27.3%, 100%), and MRI (18.2%, 81.3%). A decision-making algorithm incorporating qMRI identified progression and recommended surgery for 11 subjects (42.3%).

**Conclusions:**

Quantitative MRI shows high sensitivity to detect myelopathic progression. Our results suggest that neuroplasticity and behavioural adaptation may mask progressive SC tissue injury. qMRI appears to be a useful method to confirm subtle myelopathic progression in individual patients, representing an advance toward clinical translation of qMRI.

## Introduction

Degenerative cervical myelopathy (DCM) is among the most common causes of spinal cord (SC) dysfunction, involving age-related degeneration of the discs, ligaments, and vertebrae leading to extrinsic compression and dynamic injury.[1, 2] Low quality evidence suggests that 20%-62% of DCM subjects will deteriorate over 3–7 years.[3–5] Non-operative treatments such as cervical collars and physiotherapy are sometimes employed, but no evidence exists to support their benefit.[5] Decompressive surgery not only halts neurological deterioration, it improves outcomes and is the recommended treatment for moderate/severe DCM in recent clinical practice guidelines (CPGs).[6, 7] However, optimal management of mild DCM is controversial; surgery is a treatment option, but many patients are managed non-operatively and monitored periodically, in which case surgery is recommended if neurological deterioration occurs.[7]

Subtle progression can be difficult to identify, relying on the patient’s perception of symptoms and subjective findings of the neurological examination, while the utility of electrophysiology studies in this context has not been established.[8, 9] The majority of natural history studies have used Japanese Orthopedic Association (JOA)[10–14] or modified JOA (mJOA)[15, 16] scores as the primary outcome measure, but these have limited sensitivity and granularity, and poorly characterized reliability.[17, 18] Furthermore, these studies have used variable definitions of deterioration, including a lack of improvement (in moderate-severe subgroups),[10, 11] a 1-point decline,[15, 16] or conversion from the mild to moderate category of JOA.[12, 13] More detailed myelopathy assessments are available, but their psychometric properties and evidence supporting their use are similarly lacking.[19] As a result, practice patterns are variable and frequently rely on subjective factors, including the rudimentary question: “are your symptoms better, the same, or worse?”

An array of MRI techniques have emerged that measure aspects of SC microstructure and tissue injury.[20] Cross-sectional area (CSA) measures the degree of SC compression in DCM, and atrophy in uncompressed regions. The diffusion tensor imaging (DTI) metric fractional anisotropy (FA) reflects axonal injury and demyelination. Magnetization transfer ratio (MTR) is a more specific measure of myelin quantity. T2*-weighted imaging (T2*WI) shows strong contrast between white and grey matter, and the white matter to grey matter signal intensity ratio (T2*WI WM/GM) reflects demyelination, gliosis, calcium, and iron changes.[21, 22] We developed a clinically feasible multiparametric quantitative MRI (qMRI) protocol that collects these data across the cervical SC, producing 10 measures of tissue injury that correlate with myelopathic impairment in DCM.[21, 23]

In the current study, we compare several methods of detecting myelopathic progression, including 1) patients’ subjective impression of worsening, 2) mJOA, 3) comprehensive clinical assessments, 4) anatomical MRI, and 5) multiparametric qMRI. We hypothesize that qMRI will show a higher rate of progression than other measures due to the effects of neuroplasticity and behavioral adaption, which we suspect compensate for progressive tissue injury. Finally, we develop a practical framework for monitoring DCM patients and describe its initial implementation.

## Materials and methods

### Study design and subjects

This prospective longitudinal study received institutional approval from the University Health Network (Toronto, Ontario, Canada) and all participants provided written informed consent. A total of 58 patients were consecutively enrolled between October 2014 and August 2016 that showed one or more symptoms of cervical myelopathy (including sensory dysfunction, hand clumsiness, gait dysfunction, bladder dysfunction, or perceived weakness), one or more signs (including sensory deficit, motor deficit, hand incoordination, gait ataxia, or hyperreflexia), and imaging evidence of spinal cord compression from degenerative causes (disc, ligament, or bone). Among this cohort, 26 patients were initially managed non-operatively based on shared decision-making between the attending surgeon and patient, and this subgroup comprised the population of interest in this study. Factors that led to non-operative management were typically very mild symptoms and/or the patient’s preference to be managed non-operatively. These 26 individuals were reassessed approximately 12 months later, depending on subject availability.

### Clinical assessments

A battery of clinical assessments was administered by a clinician-scientist (ARM, 6 years experience; SKR, 10 years experience; Table 1). To reduce inter-observer variability, scripts and agreed-upon criteria to interpret answers were used. This included a modified version of the mJOA (Table 2) to simplify language and allow substitute findings, such as worsened handwriting for mild upper extremity motor impairment. The percent change in clinical measures was calculated using the maximum score as the denominator for finite scales (e.g. 18 for mJOA) or the baseline score for infinite scales (e.g. grip strength).

**Table 1:** Battery of clinical assessments for degenerative cervical myelopathy.

Clinical Measure	Description
mJOA Score[2]	18-point ordinal scale of neurological impairment including subscores for upper extremity motor function, lower extremity motor function (gait), upper extremity sensory function, and urinary function
QuickDASH[34]	44-point interval scale for upper limb function, pain, and effects on quality of life
ISNCSCI UEMS[39]	50-point interval scale for neurological function of the upper limb (power in 10 myotomes), administered separately for each upper limb
JAMAR Grip Dynamometer[32]	Measures maximal grip force in each hand; calculated as average of 3 measurements
GRASSP-M[33]	Dexterity testing of each hand to place four metallic nuts on screws, scored for precision, grasp, number of drops, and completeness (9 points), and time to completion
Monofilament Sensory Testing[40]	Semmes Weinstein monofilaments applied to C6, C7, and C8 dermatomes of each hand to measure sensation
Berg Balance Scale[41]	56-point interval scale to measure balance while standing, transferring, and performing simple tasks
GaitRITE[35]	Quantitative analysis of gait using an electronic pressure mat, measured with gait stability ratio (single stance time / double stance time)

Various clinical assessments were selected to comprehensively assess common neurological and functional impairments that occur in cervical myelopathy, including fine motor dysfunction of the hands, weakness, numbness, gait imbalance, and urinary difficulties.

**Table 2:** Modified Japanese Orthopedic Association (mJOA) score.

Category	Score	Description
Upper Extremity Motor Subscore (/5)	0	Unable to move hands
	1	Unable to eat with a spoon but able to move hands
	2	Unable to button a shirt but able to eat with a spoon
	3	Able to button a shirt with great difficulty
	4	Able to button a shirt with mild difficult OR other mild fine motor dysfunction (marked handwriting change, frequent dropping of objects, difficult clasping jewelry, etc.)
	5	Normal hand coordination
Lower Extremity Subscore (/7)	0	Complete loss of movement and sensation
	1	Complete loss of movement, some sensation present
	2	Inability to walk but some movement
	3	Able to walk on flat ground with walking aid
	4	Able to walk without walking aid, but must hold a handrail on stairs
	5	Moderate to severe walking imbalance but able to perform stairs without handrail
	6	Mild imbalance when standing OR walking
	7	Normal walking
Upper Extremity Sensory Subscore (/3)	0	Complete loss of hand sensation
	1	Severe loss of hand sensation OR pain
	2	Mild loss of hand sensation
	3	Normal hand sensation
Urinary Function Subscore (/3)	0	Inability to urinate voluntarily (requiring catheterization)
	1	Frequent urinary incontinence (more than once per month)
	2	Urinary urgency OR occasional stress incontinence (less than once per month)
	3	Normal urinary function

The mJOA is an 18 point score of functional disability specific to cervical myelopathy, including upper extremity motor subscore, lower extremity subscore, upper extremity sensory subscore, and sphincter function. The descriptions of each score are modified slightly from Benzel et al. (1991).[2]

### MRI acquisitions

All imaging was performed on the same clinical scanner (3T GE). T2-weighted imaging (T2WI) utilized sagittal FIESTA-C with 0.8 mm^3^ isotropic resolution. DTI used spin echo single shot EPI (ssEPI) with 80x80 mm^2^ field of view, anterior/posterior saturation bands, second order localized shimming with a box volume of interest, no cardiac triggering, and 1.25x1.25x5 mm^3^ resolution. MT was performed using gradient echo with and without MT prepulse with 1x1x5 mm^3^ resolution. T2*WI images used a multi-echo recombined gradient echo (MERGE) sequence with 3 echoes and 0.6x0.6x4 mm^3^ resolution. DTI, MT, and T2*WI images had 13 axial slices positioned perpendicular to the spinal cord (at C3), covering C1 to C7 using a variable gap, alternating between mid-vertebral body and intervertebral disc. Imaging time was 30–35 minutes in total, and further details of each sequence are as previously reported.[23]

### Image analysis

Images were reviewed by 2 raters (ARM, 6 years experience; AN, 6 years experience) and excluded if they showed motion or other artifacts (e.g. aliasing), along with corresponding images from the comparison examination. T2WI and T2*WI images were reviewed to identify T2WI hyperintensity and record levels with extrinsic SC compression, defined as indentation, flattening, torsion, or circumferential compression. The maximally compressed level (MCL) was subjectively determined, with discrepancies resolved by consensus. When the MCL changed between baseline and follow-up, the new level was used for comparisons.

**Fig 1:**
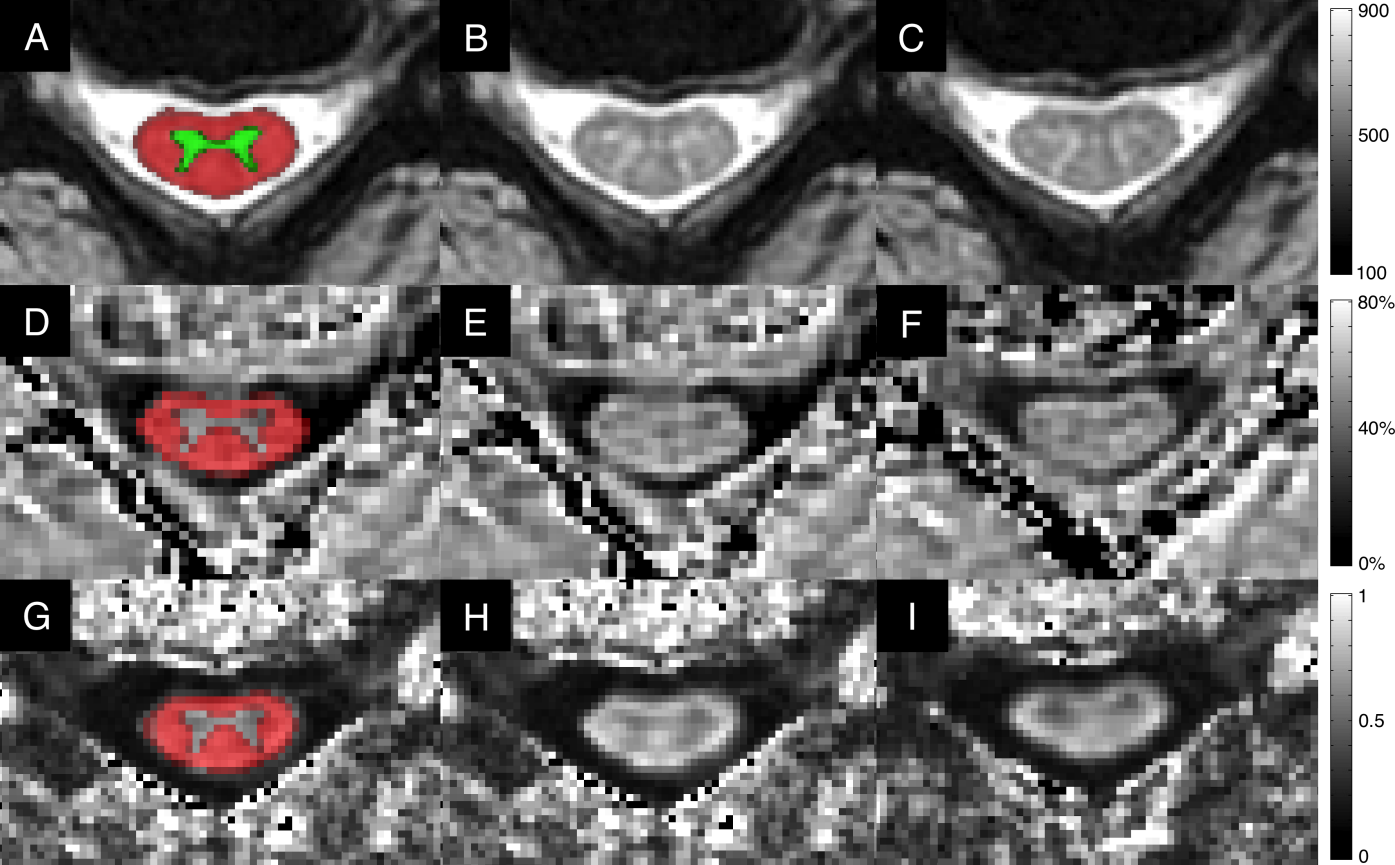
Representative images. A-C: T2*WI images showing mild spinal cord compression at C4-5, including: A) probabilistic SCT maps of GM (green) and WM (red) registered directly to anatomical images to calculate T2*WI WM/GM signal intensity ratio; B) baseline assessment; C) 13-month follow-up showing no change in the degree of spinal cord compression. Corresponding DTI FA maps (D-F) and MTR maps (G-I) are displayed demonstrating probabilistic WM maps (red, D, G); baseline assessments (E, H); and follow-up images (F, I). Visual inspection does not show obvious differences, but the quantitative readout (averaged over several slices) indicated progressive tissue injury including significant deterioration in composite score (t = -4.1), MTR of the rostral cord (z = -3.0), and T2*WI WM/GM of the rostral cord (z = -4.0). FA also showed a trend toward a decrease in the rostral cord (z = -2.3). CSA: cross-sectional area; DTI: diffusion tensor imaging; FA: fractional anisotropy; GM: grey matter; MTR: magnetization transfer ratio; T2*WI: T2*-weighted imaging; WM: white matter.

Quantitative image analysis was performed with the Spinal Cord Toolbox (SCT) v3.0 by ARM (3 years experience).[24] Automatic SC segmentation was performed, and segmentation masks were reviewed and manually corrected if necessary (Fig 1). Segmentation editing was blinded by anonymizing and randomizing baseline and follow-up scans. CSA was calculated from the T2*WI segmentation (or T2WI segmentation if T2*WI was excluded). Registration to the SCT template was performed for each dataset and FA, MTR, and T2*WI WM/GM were extracted from WM in each slice with correction for partial volume effects using the maximum a posteriori method.[25] Metrics were age-corrected based on linear regression in 40 healthy subjects (CSA: β = -0.0867 mm^2^/year, FA: β = -0.00121/year, MTR: β = -0.0815%/year, T2*WI WM/GM: β = 0.000740/year).[23] Corrected metrics were averaged across rostral (C1-C3) and caudal (C6-C7) levels, excluding compressed slices, and at MCL using a single slice for CSA or 3 slices for FA, MTR, and T2*WI WM/GM.[21] This approach produces 12 metrics, of which 10 previously demonstrated significant clinical correlations in DCM,[21] leading to exclusion of caudal CSA and MTR from this study.

### Myelopathic progression

Patients were asked if their neurological symptoms were better, the same, maybe worse (defining borderline progression), or worse (defining progression) than at the initial assessment. For mJOA, progression was defined as a decrease of ≥ 2 points and borderline progression as a 1-point decline.[18] For comprehensive clinical assessments, progression was defined as ≥ 3 measures worsening by ≥ 5%, and borderline progression as 1–2 measures worsening. qMRI progression was defined by statistical tests described in the statistical analysis section. Patients’ subjective impression was used as the clinical case definition of myelopathic progression.

### Statistical analysis

Overall α was set to 0.05. Continuous data were summarized by mean ± standard deviation (SD). Group deterioration at follow-up was analyzed with single-tailed paired t tests. 95% confidence intervals (CIs) of proportions were calculated using the Wilson method (continuity corrected). qMRI progression was tested against the null hypothesis that changes were due to measurement error (assuming normal distribution and SD = √2 * standard error of measurement, SEM), using z scores.[26] SEM of FA, MTR, and T2*WI WM/GM were derived from our previous reliability study, and SEM of CSA was calculated using T2*WI data from 5 healthy and 11 DCM subjects.[23] For rostral and caudal measures, pooled estimates of SEM were derived from healthy and DCM subjects, whereas MCL SEM values were derived from DCM subjects only (Table 3). Z scores were also averaged to yield an unweighted composite score (null hypothesis: *t* distribution, 10 degrees of freedom, d.f.s, standard error = 1/ √10). qMRI progression was defined as z score < -2.65 for any single metric or composite score: *t*_10_ < - 3.30 (p = 0.004, single-tailed, Bonferroni corrected). Sensitivity, specificity, and Youden’s Index (YI) were calculated for each measure of myelopathic progression relative to the clinical case definition (based on available follow-up events for each measure). Pairwise Fisher exact tests were used to compare the sensitivity, specificity, and concordance with the clinical case definition between measures of myelopathic progression.

**Table 3:** Summary of age-corrected Quantitative MRI metrics.

qMRI Metric	Level	Mean Difference	P Value	SEM	Individuals with Progression
CSA (mm^2^)	Rostral	-0.34 ± 1.08	0.07	0.95	0
	MCL	-3.5 ± 5.4	0.003	2.94	3
FA	Rostral	-0.027 ± 0.037	0.001	0.018	6
	MCL	-0.038 ± 0.050	0.0007	0.029	4
	Caudal	-0.016 ± 0.049	0.06	0.025	4
T2*WI WM/GM	Rostral	+0.006 ± 0.018	0.09	0.008	4
	MCL	+0.005 ± 0.039	0.21	0.034	0
	Caudal	+0.012 ± 0.033	0.03	0.022	3
MTR (%)	Rostral	-0.80 ± 3.2	0.12	1.26	2
	MCL	-1.1 ± 2.8	0.03	3.10	0
Composite Score (t_10_)		-2.2 ± 2.2	0.00004	0.316	7

Group results are displayed for the qMRI metric differences between baseline and follow-up, reported as mean ± SD. P values are reported for single-tailed paired t tests. SEM values are derived from our previous reliability study, and the SEM of CSA was measured in 5 healthy subjects and 11 DCM patients.[23] The composite score is calculated as an average of z scores for each metric, which is expected to follow a t distribution with 10 d.f.s under the null hypothesis. The number of individuals with progression detected by each measure is displayed (z < -2.65 or t_10_ < -3.30, p<0.004, one-tailed, corrected for multiple comparisons). CSA: cross-sectional area; d.f.s: degrees of freedom; FA: fractional anisotropy; MCL: maximally compressed level; MTR: magnetization transfer ratio; SEM: standard error of measurement

## Results

### Subjects

The cohort was aged 57.6 ± 9.1 years, included 15 men and 11 women, and baseline mJOA score was 15.7 ± 1.3 (21 mild, 5 moderate severity). Follow-up data included subjective impression and mJOA score for all 26 subjects (100%), anatomical MRI for 25 subjects (96.2%), and comprehensive clinical and qMRI data for 22 subjects (84.6%) (Table 4). One subject had two complete follow-up assessments due to interim subjective deterioration. Among four subjects without complete follow-up, three (11.5%) experienced rapid progression (subjectively worse, mJOA declined ≥ 2 points) requiring urgent surgery and the remaining subject reported stable symptoms and mJOA but declined follow-up.

**Table 4:** Summary of DCM patient characteristics, clinical changes, and Quantitative MRI changes at follow-up.

#	Age, Sex	mJOA	FU (m)	Subjective	mJOA	Comprehensive Clinical Assessment; Confounding Factors	Anatomical MRI	Quantitative MRI Assessment
1	56M	15	2	↓	↓	N/A	**≈**	N/A
2	52F	16	10	↓	↓	N/A	**≈**	N/A
3	60F	15	10	↓	↓	N/A	**≈**	N/A
4	47M	15	15	↓	**↓**	↓ (mJOA, R/L grip, L arm power)	↓	↓ (CSA_MCL_, FA_MCL_, FA_Caudal_)
5	50M	17	13	↑	**≈**	**≈(**L grip, L arm power); L elbow injury	**≈**	↓ (MTR_Rostral_)
6	60M	17	13	**≈**	**≈**	↓ (mJOA, R/L grip, L hand sensation)	↓	↓ (Composite, FA_Caudal_, T2*WI WM/GM_Caudal_)
7	60M	16	12	**≈**	**≈**	**≈**	**≈**	**≈**
8	69F	16	13	**≈**	**≈**	↓(L grip, Berg Balance, R hand dexterity); lumbar radiculopathy, psoriatic arthritis (hands) and knee replacement	**≈**	**≈**
9	59F	17	14	**≈**	**≈**	↓ (R grip, L arm power, R/L hand dexterity, gait stability)	**≈**	↓ (T2*WI WM/GM_Caudal_)
10	55F	15	17	↓	**≈**	↓ (R grip, L hand dexterity, gait stability); rheumatoid arthritis	↓	↓ (CSA_MCL_)
11	54F	17	14	**≈**	**≈**	**≈** (mJOA)	**≈**	**≈**
12	56F	16	12	**≈**	**≈**	**≈** (QuickDASH)	**≈**	**≈**
2^nd^ Follow-up			26	↓	**≈**	↓ (mJOA, QuickDASH, R/L grip, gait stability)	**≈**	↓ (T2*WI WM/GM_Rostral_)
13	59F	13	13	↓	**≈**	↓ (mJOA, R/L grip)	**≈**	↓ (Composite, FA_Rostral_, FA_MCL_, FA_Caudal_, MTR_Rostral_)
14	81M	17	12	**≈**	**≈**	↓ (R/L grip, L hand dexterity)	↓	↓ (Composite, FA_Rostral_, FA_MCL_
15	69M	17	13	↓	**≈**	**≈** (L grip, L hand dexterity)	**≈**	↓ (Composite, CSA_MCL_, FA_Rostral_, FA_MCL_)
16	69M	17	13	**≈**	**≈**	↓ (L grip, L arm power, L hand dexterity; L hand fasciitis	**≈**	**≈**
17	48M	14	12	↓	**≈**	**≈** (L grip, R hand sensation)	**≈**	↓ (FA_Rostral_)
18	49F	17	17	**≈**	**≈**	↓ (mJOA, QuickDASH, R/L grip); severe back pain	**≈**	**≈**
19	61M	14	13	↓	**≈**	↓ (QuickDASH, L grip, R/L sensation)	**≈**	↓ (Composite, MTR_Rostral_, T2*WI WM/GM_Rostral_)
20	61M	16	12	**≈**	**≈**	**≈** (QuickDASH)	**≈**	↓ (Composite, FA_Rostral_, T2*WI WM/GM_Rostral_)
21	58M	14	15	**≈**	**≈**	**≈**; mild TBI with post-concussion symptoms	**≈**	↓ (FA_Caudal_)
22	49M	14	11	↓	**≈**	↓ (mJOA, QuickDASH, Berg Balance	**≈**	↓ (Composite, T2*WI WM/GM_Rostral_)
23	54M	17	6	**≈**	**≈**	**≈** (L hand dexterity, gait stability)	**≈**	↓ (FA_Rostral_, T2*WI WM/GM_Caudal_)
24	54F	15	27	↑	↑	**≈(**R grip, L grip); shoulder and neck injury	**≈**	**≈**
25	45F	17	15	↑	**≈**	**≈**	↓	**≈**
26	76M	15	6	**≈**	**≈**	N/A	N/A	N/A

Subject demographics include baseline age, sex, baseline mJOA, and time to follow-up (in months). Patients subjectively rated their neurological symptoms as same/better (green), or worse (red). Change in mJOA was categorized as stable/improved (green), or declined (≥2-point decrease, red). Comprehensive clinical assessments were rated as stable (green) if < 3 clinical measures worsened by ≥5%, or declined (red) if ≥3 clinical measures worsened. Anatomical MRI was rated as declined (red) if new/worsened SC compression was present at any level, and stable (green) otherwise. Quantitative MRI was rated as stable (green) if no measures showed statistically significant worsening, or declined if any measure worsened. Subject 12 had 2 follow-up assessments, experiencing subjective deterioration after the 1^st^ follow-up. ↑ denotes improvement; **≈** denotes stability; ↓ denotes deterioration; CSA: cross-sectional area; FA: fractional anisotropy; mJOA: modified Japanese Orthopedic Association score; MTR: magnetization transfer ratio; N/A: data not available; T2*WI WM/GM: T2*-weighted imaging white matter to grey matter ratio.

### Clinical assessments

**Fig 2:**
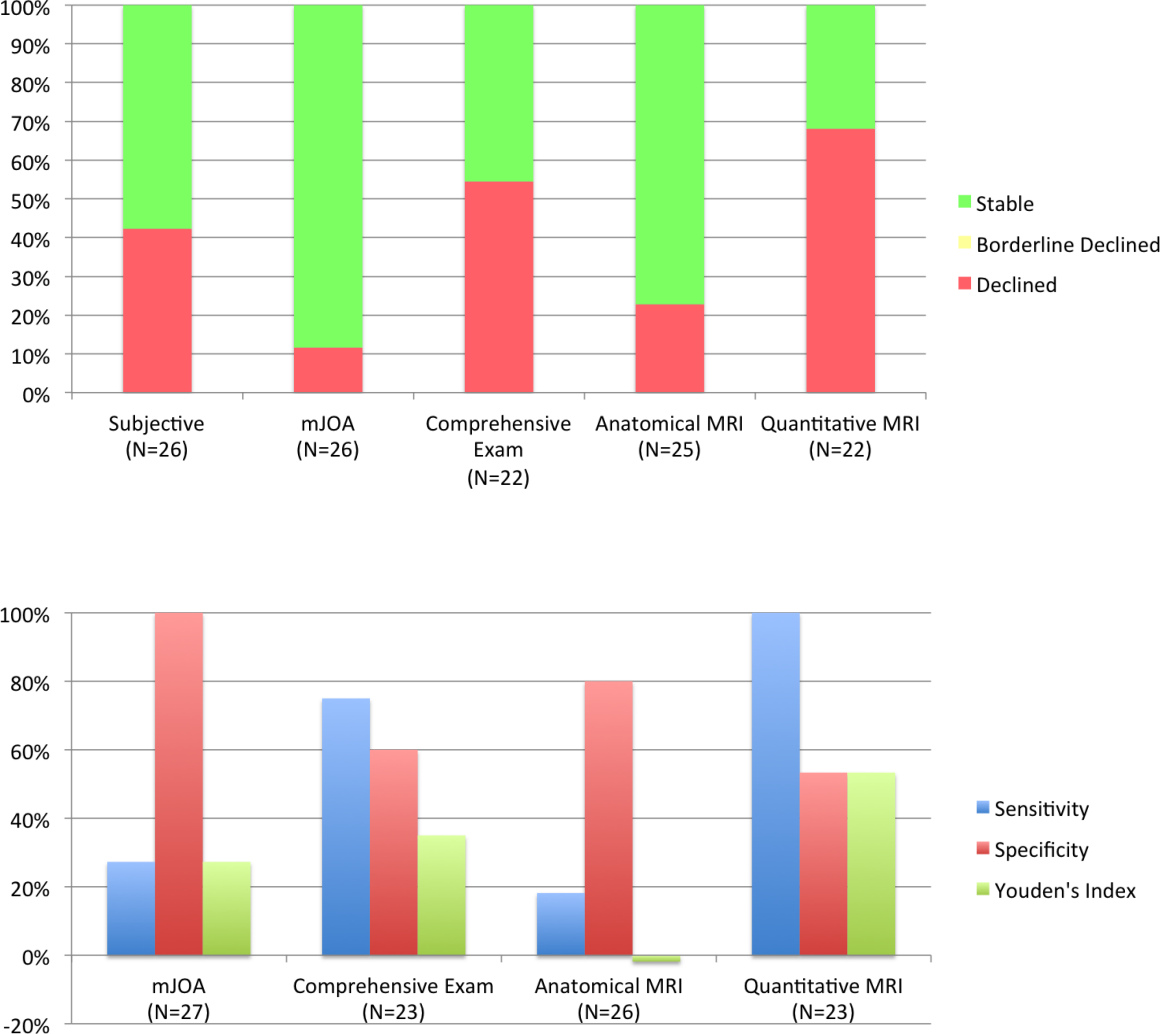
Comparison of methods to monitor for myelopathic progression in DCM. Top panel: The bar graph displays the fraction of subjects that are deemed to be stable (green), borderline declined (yellow), or declined (red) for each clinical and MRI method of monitoring. For mJOA, a 1-point decrease is considered borderline and ≥ 2-point decreases are considered declined. For comprehensive examinations, subjects that have 1 or 2 measures that worsen ≥ 5% are considered borderline and worsening of ≥ 3 measures is considered declined. For anatomical MRI, any new or increased compression that can be visually appreciated is considered declined. For qMRI, deterioration of ≥ 1 measure is considered declined. DCM: degenerative cervical myelopathy; mJOA: modified Japanese Orthopedic Association. Bottom panel: Diagnostic accuracy of each measure was measured as sensitivity, specificity, and Youden’s Index relative to patients’ subjective impression, which was selected as the clinical case definition.

Follow-up duration was 13.5 ± 4.9 months (range 6–27). Eleven patients (42.3%, 95% CI: 24.0%-62.8%) reported subjective neurological worsening at latest follow-up, and 15 (57.7%, 95% CI: 37.2%-76%) reported feeling the same or better (Fig 2, Table 4). Using mJOA, 3 subjects (11.5%, 95% CI: 3.0%-31.3%) showed clear progression. In comparison with the gold standard (subjective worsening), mJOA detected progression in 3/11 follow-up events (sensitivity = 27.3%, specificity = 100%, YI = 27.3%).

Among 22 subjects with complete follow-up data, comprehensive clinical assessments identified progression in 12 subjects (54.5%, 95% CI: 32.7%-74.9%), including 6/8 follow-up events with subjective deterioration (sensitivity = 75%, specificity = 60%, YI = 35%). Abnormal results included grip strength (15 subjects, 7 bilateral), hand dexterity (7 subjects, 1 bilateral), mJOA (7), QuickDASH (6), gait stability ratio (5), arm power (4 subjects), sensation (3 subjects, 1 bilateral), and Berg Balance scale (2). Seven subjects (31.8%) had physical injuries/conditions that potentially affected follow-up clinical assessments (e.g. recent hip replacement affecting walking assessment).

### Anatomical imaging

Baseline anatomical images showed spinal cord compression at a total of 79 intervertebral levels (3.0 levels/subject), with 21/26 subjects having multilevel SC compression. T2WI hyperintensity was present in 14/26 subjects. At follow-up, two subjects had new SC compression (total: 3 levels) and compression resolved at one level in another subject. The degree of cord compression also increased three subjects (total: 4 levels). No changes in T2WI hyperintensity were observed. Overall, five subjects had progression on anatomical imaging (20%, 95% CI: 7.6%-41.3%), including 2/11 events with subjective progression (sensitivity = 18.2%, specificity = 80%, YI = -2%).

### Quantitative MRI

All DTI and MT datasets were of acceptable quality, but two T2*WI datasets were degraded by motion artifact and excluded. Individual slices were excluded 24/585 DTI, 17/585 MT, and 11/533 T2*WI images. Analysis was successful for all remaining data, including accurate registration to the SCT atlas.

At the group level, all age-corrected qMRI metrics deviated pathologically at follow-up, including significant changes in five measures (CSA_MCL_, FA_Rostral_, FA_MCL_, T2*WI WM/GM_Caudal_, and MTR_MCL_) and trends in three (CSA_Rostral_, FA_Caudal_, T2*WI WM/GM_Rostral_(Table 3). Composite score showed the strongest group change (p = 0.00004).

**Fig 3:**
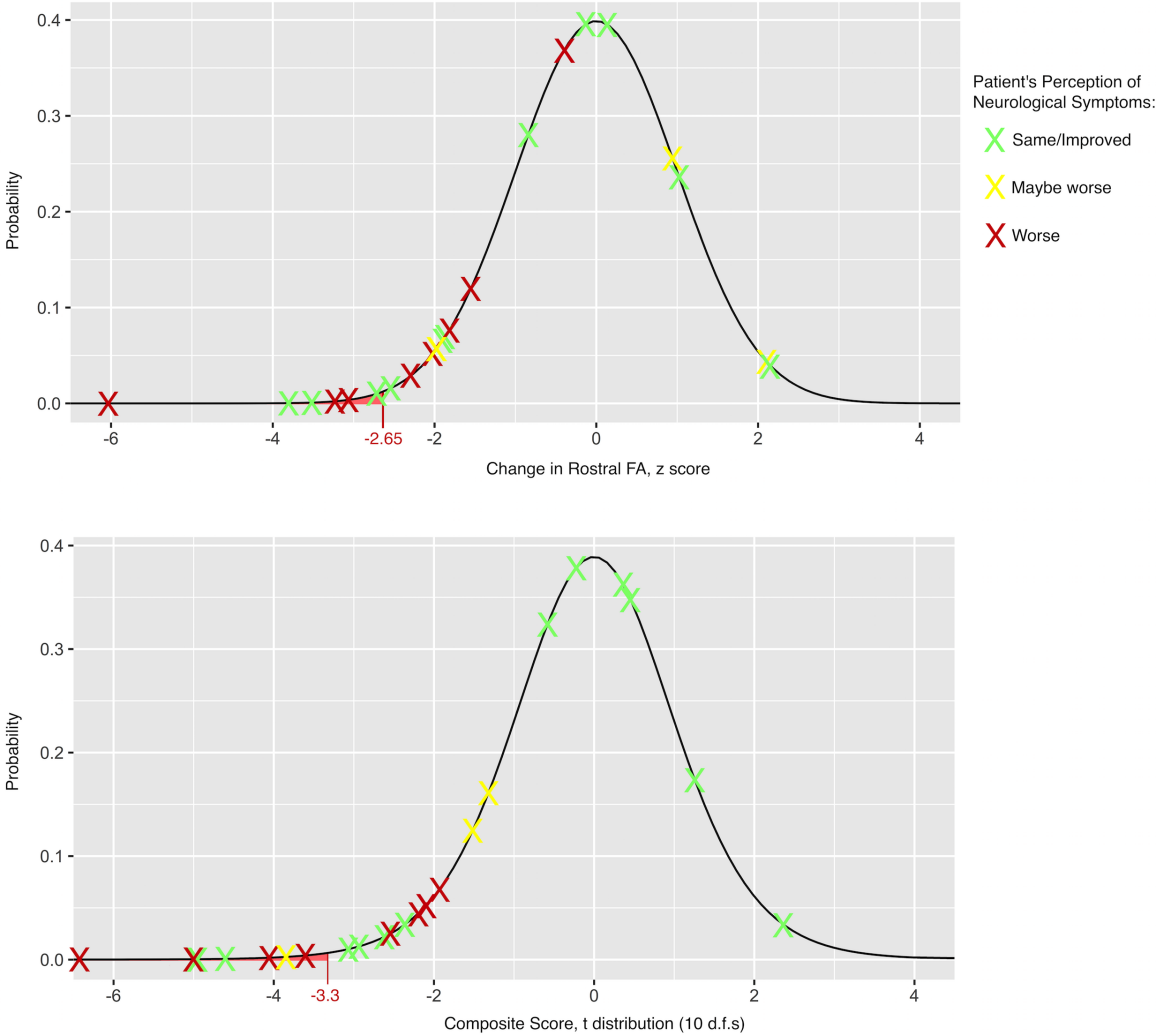
Distribution of observed changes in Quantitative MRI (qMRI) metrics at follow-up. The observed changes in age-corrected qMRI metrics for individual subjects (displayed as Xs) are plotted in relation to the expected distribution based on the null hypothesis of no change, using test-retest reliability data to characterize the SEM and calculate z scores. The results for FA_Rostral_ (top panel) are overlaid on a normal distribution. The composite score is calculated as an average of z scores for each metric, which is overlaid on a t distribution with 10 d.f.s (bottom panel). Each result is colour-coded based on the patient’s subjective impression of neurological worsening (red: worse, yellow: maybe worse, and green: the same or better). CSA: cross-sectional area; d.f.s: degrees of freedom; FA: fractional anisotropy; MCL: maximally compressed level; PDF: probability density function, SEM: standard error of measurement.

In individual patients, qMRI progression occurred in 15/22 (68.2%, 95% CI: 45.1%-85.3%) (Fig 3, Table 4). FA_Rostral_ was the most sensitive single qMRI measure, identifying progression in six subjects, while seven subjects deteriorated on composite score. All eight follow-up events with subjective worsening were detected by qMRI (sensitivity = 100%, specificity = 53.3%). qMRI rarely showed statistical improvements (potential outliers), occurring in 2/247 comparisons (0.8%).

### Comparison of approaches to monitor myelopathic progression

qMRI showed greater sensitivity to detect myelopathic progression compared with mJOA (p = 0.003) and anatomical MRI (p<0.001), but not compared with comprehensive clinical assessments (p = 0.47). mJOA had higher specificity for progression than qMRI (p = 0.002) and comprehensive clinical assessments (p = 0.007), but not anatomical MRI (p = 0.10). qMRI showed a trend toward higher concordance with the gold standard (subjective worsening) compared with anatomical MRI (p = 0.08), whereas differences with mJOA (p = 0.55) and comprehensive clinical assessments (p = 0.75) were non-significant.

### Clinical implementation

**Fig 4:**
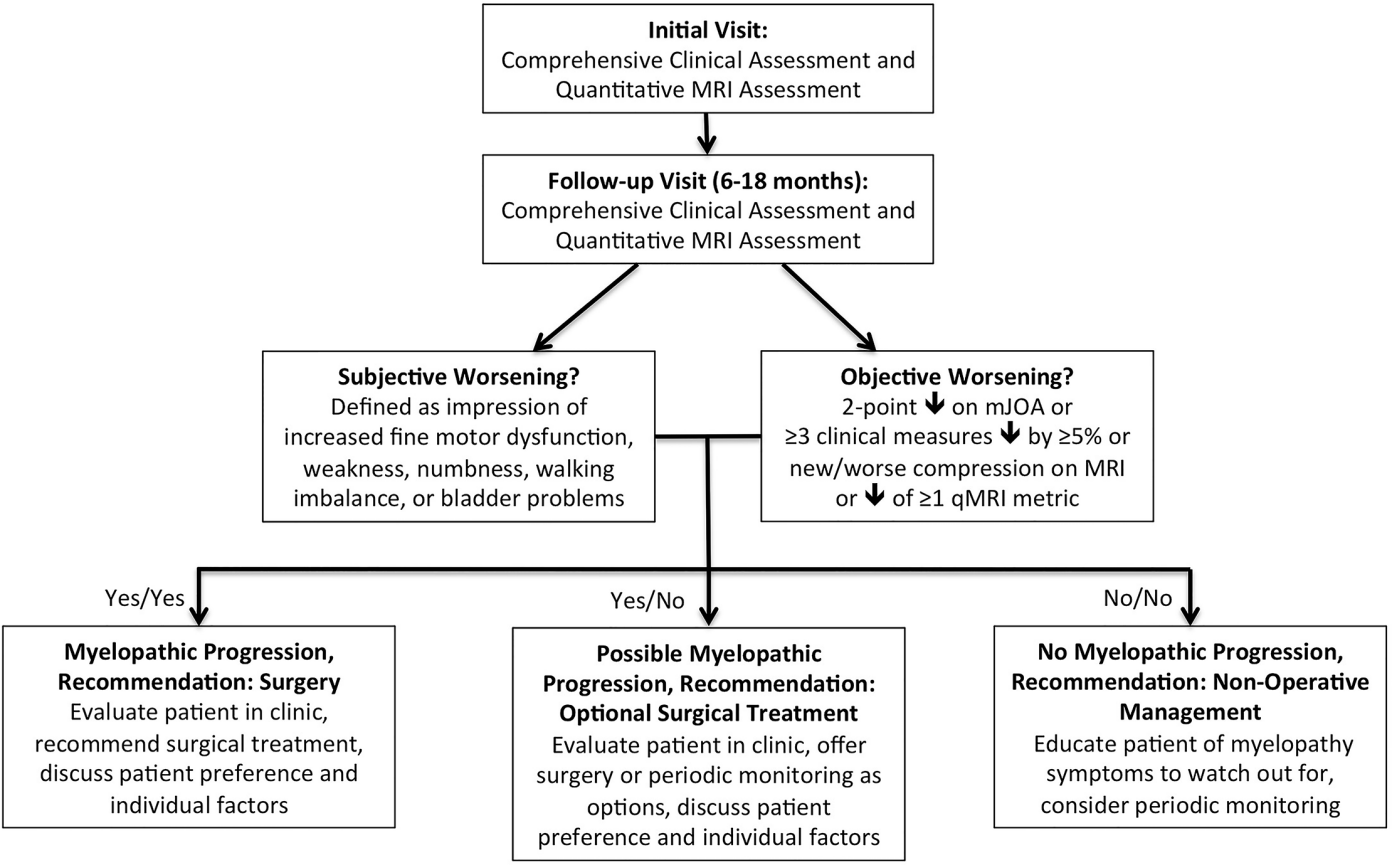
Decision-making algorithm for degenerative cervical myelopathy patients initially managed non-operatively. The decision-making algorithm requires clinical and quantitative MRI data collection at 2 time-points, and takes into account the patient’s subjective impression of worsening and objective measures of progression, including mJOA, a battery of clinical assessments, anatomical MRI, or quantitative MRI.

Based on the results, the authors developed a practical definition of myelopathic progression: subjective progression of neurological symptoms and any objective sign of progression, with the latter including mJOA, comprehensive clinical assessments, anatomical MRI, or qMRI. (Fig 4). Possible myelopathic progression was defined as either subjective or objective worsening. Using these definitions, 11 subjects had progression at latest follow-up (42.3%, 95% CI: 24.0%-62.8%), seven (30.8%) had possible progression, and eight were stable (including three with clinical deterioration that was attributed to another cause). Fifteen subjects were invited for reassessment in clinic, with the decision-making algorithm being used to help guide surgical recommendations, in addition to patient-specific factors such as preferences and goals. The remaining subjects were educated about myelopathy symptoms and encouraged to contact their surgeon if subjective progression occurred. To date, seven patients have been reassessed in clinic and two are planned for operative treatment, two have pending visits, and six declined, stating they are comfortable monitoring their symptoms.

## Discussion

In this study, myelopathic progression was more frequently detected with multiparametric qMRI than any other method. Furthermore, qMRI progression was highly congruent with subjective progression, indicating that the macro- and microstructural changes captured by qMRI are clinically meaningful. qMRI demonstrated greater sensitivity for progression than anatomical MRI and mJOA, but it should be noted that it was less specific than these measures. This is possibly explained by the concept of neuroplasticity and/or behavioural adaptation, as qMRI may pick up subtle progressive damage that an individual can compensate for (discussed below). qMRI also showed the highest concordance with subjective progression, although the differences with other measures were non-significant and no conclusions can be drawn about superiority. At present, a “gold standard” method of determining neurological progression in patients with DCM does not exist, and thus the ground truth is unknown. As a result, clinical practice varies widely, and many surgeons rely on patients’ subjective impression of neurological deterioration as a trigger to recommend surgery. Detailed clinical assessments showed moderate sensitivity and specificity to detect deterioration, while anatomical MRI was insensitive but fairly specific. Overall, we felt that all of the methods of determining myelopathic progression had potential clinical utility, and we incorporated them into a decision-making algorithm that is consistent with recent CPGs that recommend surgery when myelopathic progression occurs.[7] qMRI results were sufficiently convincing to include in this algorithm, providing confirmatory evidence of deterioration. However, caution is warranted before qMRI results take a more central role in decision-making, as this study had a relatively small sample size. In fact, our proposed algorithm is arguably more conservative than the decision-making process currently used by many surgeons, as stable qMRI results helped to avoid surgery in 2 patients that had concerning findings at follow-up that were difficult to interpret (fluctuating symptoms and confounding physical ailments). Ultimately, the algorithm provides only general guidance and the final decision regarding surgery occurs in a standard clinic visit that incorporates patient preference and other factors, including a fulsome discussion to balance risks and benefits and select the optimal treatment. The initial implementation of this algorithm has led to surgical treatment in two patients; both showed only 1-point decreases in mJOA and minimal neurological worsening, which some surgeons would manage conservatively, but qMRI helped to confirm progression. This study represents, to the authors’ knowledge, the first instance in which qMRI measurement of SC integrity has been used to inform decision-making in individual patients, constituting an important step toward clinical translation. Longitudinal monitoring for progression is an attractive first use of qMRI because it circumvents the normal inter-subject variability of these data, which limit qMRI’s utility for diagnosis and prognostication.[23, 27] However, further data, including long-term clinical outcomes, are needed to fully characterize the utility of this approach.

Eight qMRI metrics demonstrated significant deterioration in either group or individual analyses, with the greatest individual and group differences observed using the composite score. The composite averages data from the compressed cord and uncompressed regions above and below, which we feel strikes a good balance between greater sensitivity to pathology (using measurements at the level of compression),[21] and unbiased values from undistorted regions.[28] Calculation of an unweighted composite score is a naïve approach that could be further strengthened by using weightings (e.g. logistic regression), but this was not performed to avoid overfitting given our small sample. Other groups have also developed multiparametric protocols,[29–31] and our data suggest that this type of approach can overcome the limitations of single qMRI techniques, such as modest reliability. Two potential outliers (improvements of z > 2.65) were observed, close to the expected value of 1.1, validating our statistical approach. These changes may represent tissue regeneration (e.g. remyelination), or alternatively these and some qMRI decreases could be spurious due to sampling error, artifacts, analysis errors, or inaccurate estimation of SEM.

Our results suggest that DCM is less benign than previously thought.[4] mJOA showed a rate of progression of 3.0%-31.3%, consistent with previous reports (adjusting for follow-up duration).[10–16] In contrast, progression with our clinical battery was 32.7%-74.9%, in spite of missing follow-up data in three subjects that had rapid deterioration. The higher rate of progression using more comprehensive methods was expected, as our clinical instruments were selected to detect subtle myelopathic changes.[19] Quantitative MRI showed even higher frequency of progression (40.8%-82.0%). These results cast doubt that the natural history of myelopathy has been accurately characterized, and larger prospective studies are needed with clear definitions of progression and comprehensive assessments. If the natural history is in fact as aggressive as our estimates suggest, it may be advisable to recommend surgery for patients with mild DCM. However, further research is needed to determine the impact of subtle progression on 1) quality of life and 2) the risk of more substantial deterioration before CPGs are modified for mild DCM. Long-term monitoring of non-operative subjects will also reveal if isolated qMRI progression is a precursor to physical deterioration.

Myelopathy can present variably, and the design of valid, reliable, and responsive instruments is challenging. Unfortunately, assessments that rely on patients’ perception of progression are prone to recall bias and numerous other factors such as personality and anxiety. One subject clearly had worsened gait and hand dexterity but reported feeling “the same”, highlighting that patients are often unaware or resistant to acknowledge neurological deterioration. Therefore, it is important to establish accurate and reliable clinical methods of confirming myelopathic progression to advise surgical decision-making. The mJOA is easy to administer and provides a useful summary measure, but lacks sensitivity to detect subtle changes.[6] Furthermore, one-point changes in mJOA are probably not trustworthy, based on one small reliability study.[18] Two-point mJOA changes were specific but not sensitive for progression, and thus, we feel that mJOA is not adequate as a standalone measure for detecting progression. Comprehensive clinical assessments were far more sensitive but less specific, primarily due to confounding physical ailments that commonly affect older individuals. The neurological impairments in cervical myelopathy include gait imbalance, hand incoordination, sensory dysfunction, weakness (e.g. hand intrinsics), and bladder dysfunction, which are all captured in our comprehensive clinical assessments. Grip strength was the most sensitive measure of progression, which has high inter-subject variability but excellent within-subject reliability, making it ideal for longitudinal monitoring.[32] Decreases in hand dexterity were also often encountered, which involved judging subjects’ precision, grasp, and speed of tightening metallic nuts on screws.[33] QuickDASH, a questionnaire of upper limb function,[34] frequently showed progression, but it is not specific to myelopathic impairment. Gait impairment in DCM primarily involves imbalance, which is difficult to measure, and quantitative analysis with GAITRite may offer greater sensitivity than the 30-meter walk test.[35] However, quantitative gait analysis produces dozens of parameters, and further investigation is needed to determine if gait stability ratio is the optimal measure. Quantitative standardized clinical assessments are needed to enable precise quantification of myelopathic impairment (i.e. “personalized medicine”), which will allow more informed treatment decisions and greater standardization of care. However, direct measurement of spinal cord integrity with qMRI is also appealing because it avoids the challenges of clinical measurement, which assess injury to the SC indirectly. Further investigation of multimodal electrophysiology approaches, including motor and sensory evoked potentials and contact head evoked potentials (CHEPs), may also prove useful.[36] Moving forward, we envision that DCM management will evolve such that it is driven by a combination of quantitative clinical, electrophysiological, and qMRI assessments.

qMRI showed a higher rate of progression than clinical measures, suggesting that homeostatic mechanisms act to preserve normal function in the context of progressive tissue injury. Physical assessments (strength, dexterity) showed higher rates of progression than self-reported functional measures (mJOA, QuickDASH), which may be related to behavioural adaption, recall bias, and psychological denial of symptom progression. DCM patients typically alter their grasp and gait, often unconsciously, to maintain function despite incoordination and hyperactive reflexes. Furthermore, deterioration of low-level physical functions (e.g. grip strength) occurred more often than higher-level functions (gait, dexterity) that involve more complex neurological systems, potentially due to neuroplasticity.[37, 38] Complex neural circuits show more plasticity than simple circuits, such as spinal reflexes, due to the number of neurons and synapses involved.[37] However, our data are only suggestive of this concept; histopathological studies that correlate qMRI measurements with actual tissue changes are needed to fully elucidate these mechanisms. However, other qMRI techniques such as functional MRI have provided similar evidence of neuroplasticity in spinal cord injury (SCI) and may yield further insights as they become more refined.[38]

This study was subject to several limitations including a relatively small sample size and a lack of electrophysiology data, and larger multi-center confirmatory studies are planned to validate our results and more accurately characterize test-retest reliability, relationships with age, and the natural history of DCM. The cohort of 26 patients may be subject to selection bias, as the decision to manage patients non-operatively is subjective and varies between surgeons. The effect of age on qMRI metrics was derived from a cross-sectional study of 40 healthy subjects, but a longitudinal study in this population may be beneficial to fully elucidate the effects of age. Four subjects (15%) did not have complete follow-up data available, but were included in certain analyses to determine the rate of myelopathic progression. This may have resulted in underestimates of the rate of progression and sensitivity of qMRI and comprehensive clinical assessments because three of these subjects had severe worsening and would likely have shown worsening on all measures (extrapolating from our other results). The accuracy of CSA measurement could likely be improved with high-resolution T2WI using a different sequence that is less affected by motion. DTI with cardiac triggering may slightly improve reliability, based on previous data.[23] We assumed that qMRI measurement errors were normally distributed, but this is potentially incorrect. The definitions of myelopathic progression on mJOA (2-points) and comprehensive clinical assessments (3 measures by 5%) were arbitrary, and greater research is needed to optimize clinical assessments for myelopathic deterioration. The methods used in this study require considerable resources (MRI, clinical tools, expertise) that may not be feasible to implement in some clinical settings, highlighting the importance of developing and validating simple clinical tools. Finally, our decision-making algorithm is an initial attempt at rational use of these novel assessments, but this is expected to evolve as greater experience is obtained, while taking into account additional patient-specific factors.

## Conclusions

Multiparametric qMRI appears to detect subtle myelopathic progression with high sensitivity in individual DCM patients, while correlating well with patients’ perceptions. This novel approach may be a useful adjunctive method to confirm neurological worsening, warranting further study with long-term follow-up. The natural history of DCM appears to be more progressive than previously thought, perhaps because neuroplasticity and behavioral adaption act to mask progressive tissue injury. Our pilot implementation of qMRI into a decision-making algorithm represents one of the first clinical uses of SC qMRI to inform management of individual patients.

## Abbreviations

CPGs: clinical practice guidelines
CSA: cross-sectional area
DCM: degenerative cervical myelopathy
DTI: diffusion tensor imaging
FA: fractional anisotropy
GE: General Electric
GM: grey matter
GRASSP-M: graded redefined assessment of strength, sensibility, and prehension for myelopathy
ISNCSCI: International Standard for Neurological Classification of Spinal Cord Injury
JOA: Japanese Orthopedic Association
MCL: maximally compressed level
mJOA: modified Japanese Orthopedic Association score
MRI: magnetic resonance imaging
MT: magnetization transfer
MTR: MT ratio
qMRI: quantitative MRI
SC: spinal cord
SCI: spinal cord injury
SCT: Spinal Cord Toolbox
SEM: standard error of measurement
T2WI: T2-weighted imaging
T2*WI: T2*-weighted imaging
T2*WI WM/GM: T2*-weighted imaging white matter to grey matter signal intensity ratio
UEMS: upper extremity motor score
WM: white matter
