# Correction: Monitoring for myelopathic progression with multiparametric quantitative MRI

**DOI:** 10.1371/journal.pone.0204082

**Published:** 2018-09-12

**Authors:** Allan R. Martin, Benjamin De Leener, Julien Cohen-Adad, Sukhvinder Kalsi-Ryan, David W. Cadotte, Jefferson R. Wilson, Lindsay Tetreault, Aria Nouri, Adrian Crawley, David J. Mikulis, Howard Ginsberg, Eric M. Massicotte, Michael G. Fehlings

The affiliation for the fifth author is incorrect. David W. Cadotte is affiliated with University of Calgary, Calgary, Alberta, Canada.
